# Metabolic acidosis is associated with increased risk of adverse kidney outcomes and mortality in patients with non-dialysis dependent chronic kidney disease: an observational cohort study

**DOI:** 10.1186/s12882-021-02385-z

**Published:** 2021-05-19

**Authors:** Navdeep Tangri, Nancy L. Reaven, Susan E. Funk, Thomas W. Ferguson, David Collister, Vandana Mathur

**Affiliations:** 1 Department of Internal Medicine, Rady Faculty of Health Sciences, Max Rady College of Medicine, University of Manitoba, Seven Oaks General Hospital, Manitoba R2V 3M3 Winnipeg, Canada; 2 Strategic Health Resources, La Cañada, California USA; 3 Division of Nephrology, Department of Medicine, McMaster University, Hamilton, Ontario Canada; 4 MathurConsulting, California Woodside, USA

**Keywords:** Chronic kidney disease, observational study, metabolic acidosis, serum bicarbonate, CKD progression, renal replacement therapy, dialysis, mortality, transplantation

## Abstract

**Background:**

Management of chronic kidney disease (CKD) requires the management of risk factors, such as hypertension and albuminuria, that affect CKD progression. Identification of additional modifiable risk factors is necessary to develop new treatment strategies for CKD. We sought to quantify the association of metabolic acidosis with CKD progression and mortality in a large U.S. community-based cohort.

**Methods:**

In this longitudinal, retrospective cohort study we identified non-dialysis-dependent patients with stage 3‒5 CKD from Optum’s de-identified integrated electronic health records. We selected cohorts of patients with confirmed metabolic acidosis or normal serum bicarbonate levels based on 2 consecutive serum bicarbonate values: 12 to < 22 mEq/L or 22-29 mEq/L, respectively, 28‒365 days apart. The primary composite outcome was ≥ 40 % decline in estimated glomerular filtration rate (eGFR), renal replacement therapy (chronic dialysis or kidney transplant), or all-cause mortality (DD40). Secondary outcomes included each component of the composite outcome. Cox proportional hazards models were used for the DD40 outcome and secondary outcomes, while logistic regression models were used for the DD40 outcome at 2 years.

**Results:**

A total of 51,558 patients qualified for the study. The unadjusted 2-year incidence of adverse renal and fatal outcomes was significantly worse among patients in the metabolic acidosis group vs. those who had normal serum bicarbonate levels: 48 % vs. 17 % for DD40, 10 % vs. 4 % for ≥ 40 % decline in eGFR, 20 % vs. 6 % for renal replacement therapy, and 31 % vs. 10 % for all-cause mortality (all *P* < 0.001). Over a ≤ 10-year period, for each 1-mEq/L increase in serum bicarbonate, the adjusted hazard ratio for DD40 was 0.926 (95 % confidence interval [CI], 0.922–0.930; *P* < 0.001); over a ≤ 2-year period, the adjusted odds ratio for DD40 was 0.873 (95 % CI, 0.866–0.879; *P* < 0.001).

**Conclusions:**

In this large community cohort of patients with stage 3‒5 CKD, the presence of metabolic acidosis was a significant, independent risk factor for the composite adverse outcome of CKD progression, renal replacement therapy, and all-cause mortality (DD40).

**Supplementary Information:**

The online version contains supplementary material available at 10.1186/s12882-021-02385-z.

## Background

Chronic kidney disease (CKD) is a major public health problem. Approximately 14 % of the general population has CKD and more than 117,000 patients per year initiate dialysis or require a kidney transplant [1]. The mortality rate among patients with CKD (117.9 per 1000 patient-years) is more than double that of people without CKD (47.5 per 1000 patient-years) [2].

Metabolic acidosis is a common complication of advanced CKD and is caused by a combination of dietary and metabolic acid load and diminished net acid excretion [3]. In patients with CKD, metabolic acidosis is associated with several adverse outcomes, including progressive CKD, cardiovascular events, impaired immune response, bone and muscle loss, and death [4, 5].

Previous studies have examined the association between metabolic acidosis and adverse kidney outcomes, with conflicting results. In several observational studies there was an association between serum bicarbonate and CKD progression (defined as end-stage kidney disease requiring dialysis or transplant), a reduction of 50 % in estimated glomerular filtration rate (eGFR), or reaching an eGFR of less than 15 mL/min/1.73 m^2^ [6, 7], but in other studies, there was no association after adjustment for baseline eGFR [8]. These studies had several limitations, including single serum bicarbonate measurements [6, 8] and evaluation of patient populations from single centers, a small number of sites, or clinical trial samples that may not be representative of real-world patient populations [8–11].

Here we report findings from a cohort study of more than 51,000 patients with CKD with or without metabolic acidosis derived from a real-word, validated, electronic medical record dataset with a follow-up period of up to 10 years. Our objective was to determine if metabolic acidosis is an independent risk factor for adverse renal outcomes and death, defined as the composite of a ≥ 40 % decline in eGFR, renal replacement therapy (chronic dialysis or kidney transplant), or all-cause mortality (DD40), and to quantify the magnitude of its effect [12].

## Methods

### Study design and data sources

We conducted an observational, retrospective cohort study of United States (U.S.) patients with CKD and available serum bicarbonate measurements who either had at least 2 years of longitudinal follow-up or died during the 2-year period. Data from 1 to 2007 through 31 March 2017 was extracted from the Optum’s de-identified Integrated Electronic Health Record (EHR) Database; all data are de-identified in compliance with the Health Insurance Portability and Accountability Act (HIPAA), so consent was not required. The Optum database is a longitudinal clinical repository that includes 81 million insured and uninsured patients from several large healthcare provider organizations in all 50 U.S. states and Puerto Rico [13]. Extracted data elements were derived from inpatient and outpatient EHRs and administrative systems, including information on laboratory results, medication prescriptions, coded diagnoses and procedures, and provider notes extracted by natural language processing. Data cleaning included application of validity parameters to reported laboratory values and exclusion of patients with death dates prior to 2007 (Additional file 1).

Data extraction incorporated a unique patient identifier linking EHR data to a single-insurer claims database of commercial and Medicare Advantage plans. Claims data were available for 9.3 % of patients over the selected date range. Linked claims data were used to validate the definition of chronic dialysis used for the EHR data analyses (Additional file 2).

### Study cohort

The study cohort was selected from a database extract consisting of patients who had at least 1 year of EHR activity with at least 3 eGFR results of < 60 ml/min/1.73 m^2^ and at least 3 serum bicarbonate results with at least 1 value between 12 and 29 mEq/L. Inclusion in the study cohort required patients to have 2 consecutive valid serum bicarbonate values 28 and 365 days apart that were either between 12 and < 22 mEq/L (metabolic acidosis) or between 22 and 29 mEq/L (range of normal serum bicarbonate). The first of the 2 serum bicarbonate values was considered the baseline serum bicarbonate value, and the date of the test was designated the index date. Inclusion also required a baseline eGFR value of between > 10 and < 60 ml/min/1.73 m^2^. Baseline eGFR was calculated using the CKD-EPI (Chronic Kidney Disease Epidemiology Collaboration) equation [14] as the mean of eGFR values from the 90 days preceding the last eGFR test on or before the index date. Serum bicarbonate and eGFR values that were collected during hospital inpatient admissions or emergency department visits with a concurrent diagnosis code for acute kidney injury were excluded because they could not be considered representative of a chronic condition. In addition, patients were required to have at least 1 year of pre-index activity in the EHR (clinic visit, healthcare facility encounter, or laboratory test) and at least 2 years of post-index activity, unless the patient died within the 2-year outcome period. Patients were excluded if they had any pre-index evidence of chronic dialysis or kidney transplantation (diagnosis or procedure code, or outpatient eGFR result ≤ 10 mEq/L).

To ensure an adequate sample size for the metabolic acidosis group, an iterative patient selection algorithm was used to oversample patients with serum bicarbonate values between 12 and < 22 mEq/L. Specifically, the selection algorithm examined records beginning after 1 year of patient activity and initially searched for a qualifying pair of consecutive serum bicarbonate values between 12 and < 22 mEq/L before examining bicarbonate values between 22 and 29 mEq/L.

For statistical modeling, the primary analysis cohort was defined as patients meeting inclusion criteria without missing data. Missing data were a consideration only for laboratory values and were not imputed. The primary analysis cohort excluded patients who had missing albumin-to-creatinine ratio (ACR) data (3,781 [22 %] of patients with metabolic acidosis and 15,770 [46 %] of patients with normal serum bicarbonate) (Additional file 3).

### Variables

The primary exposure variable was baseline serum bicarbonate. Additional demographic and clinical variables known or hypothesized to be associated with CKD progression were assessed on the index date and included age, sex, race, diabetes, hypertension, heart failure, comorbidity burden (measured by the Charlson Comorbidity Index [CCI]), baseline eGFR, and baseline log urine albumin-creatinine ratio (ACR) [11, 15].

Age was assessed on the index date by birth year, with the caveat that persons born in 1928 or earlier were assigned 1928 as a birth year to ensure HIPAA compliance. Baseline comorbidities were assessed by a single occurrence of any relevant diagnosis code in all available pre-index data. Log ACR was defined as the closest laboratory value on or before the index date.

A complete list of variable definitions, data sources, and conversions is provided in Additional file 1; diagnosis codes for comorbidities and outcomes are provided in Additional file 3.

### Outcomes

The primary outcome assessed in this study was the composite end point of a decline in eGFR ≥ 40 %, renal replacement therapy (RRT; chronic dialysis or kidney transplant), or all-cause mortality, referred to as “DD40.” A ≥ 40 % or greater decline in eGFR was included as a component of the composite end point because it has been recently validated and accepted by regulatory authorities as an acceptable outcome to define CKD progression [12, 16]. Each component of DD40 was evaluated separately as secondary outcomes. A kidney-specific composite outcome of RRT or a decline in eGFR ≥ 40 % (RRT40), and RRT alone were also evaluated as secondary outcomes.

Death was identified by month and year through a linkage to Social Security data by Social Security number prior to data de-identification. Where the death date was missing for an identified death, the patient’s last confirmed interaction with the healthcare provider was assumed as the date of death.

Initiation of chronic dialysis required development of a multifactorial definition because most outpatient dialysis care in the United States (U.S.) is delivered by specialty providers that submit claims to insurers but do not systematically share EHR data. For this study, chronic dialysis was identified at first occurrence in the medical record of a nonemergent outpatient eGFR test result ≤ 9 mL/min/1.73 m^2^, an International Classification of Diseases, Ninth Revision, Clinical Modification (ICD-9-CM) diagnosis code of 585.6, an ICD-10-CM diagnosis code of N18.6, or a diagnosis or procedure code indicating a dialysis encounter or procedure (Additional file 2). This definition of dialysis was validated in a patient subset that had concurrent EHR data and medical insurance claims that included services by outpatient dialysis providers. Renal transplant was identified by procedure code, ICD-9-CM/ICD-10-CM diagnosis code or inpatient diagnosis-related group (DRG) code (Additional file 3).

In unadjusted analyses and 2-year statistical models, decline in eGFR was assessed by comparison of eGFR at 2 years to baseline eGFR. Similar to the definition of baseline eGFR, the eGFR at 2 years was defined as the mean of eGFR values during 90 days before the final eGFR result in the 2-year outcome period, or prior to death, dialysis, or transplantation if these occurred earlier. For statistical models using all available data (maximum 10 years, median 3.9 years), eGFR decline was identified upon the first occurrence of an eGFR value that when averaged with eGFR values during the prior 90 days represented a ≥ 40 % decline from baseline eGFR.

### Statistical analysis

Baseline patient characteristics were compared between the metabolic acidosis and normal serum bicarbonate groups using the chi-squared test, *t*-test, or 2-tailed Wilcoxon rank-sum test, as appropriate. The 2-year incidence of DD40 and individual component outcomes were compared between the 2 groups in the total cohort and within subgroups by CKD stage using the chi-squared test. Cox proportional hazards models were used for the DD40 outcome and secondary outcomes of RRT40, RRT, and all-cause mortality in all available post-index data, while logistic regression models were used for the DD40 outcome at 2 years. In both statistical models, serum bicarbonate was the primary exposure variable, evaluated as a continuous variable, with adjustments for the following demographic and clinical characteristics known or hypothesized to be associated with CKD progression. Modeled covariates were structured as follows: 1-mEq/L increase in serum bicarbonate, 1-year increase in age, 1-mL/min/1.73 m^2^ increase in eGFR, and log 1-mg/g increase in ACR as continuous variables; comorbidities (diabetes, hypertension, heart failure) as dichotomous variables; and sex, race, and CCI score (0, 1, 2, ≥ 3) as a categorical variables.

For Cox proportional hazards models of DD40 and all-cause mortality, patients who did not experience the outcome were censored at their last interaction with the health system or upon reaching March 31, 2017. For Cox proportional hazards models of RRT and RRT40, patients were also censored at time of death.

#### Subgroup and sensitivity analyses

Cox proportional hazards models for DD40 were performed in subgroups of patients ≥ 65 and < 65 years of age, as well as the extended cohort that included patients who had missing values for urine ACR. We also evaluated decline in eGFR using an alternative definition (the first single outpatient eGFR value representing a decline of ≥ 40 % from baseline eGFR) in sensitivity analyses of DD40 and RRT40. The effect of oversampling for metabolic acidosis was evaluated by reconstructing the patient cohort without oversampling, in which the effect of serum bicarbonate on DD40, RRT40, RRT, and death was evaluated in Cox proportional hazards models with the same predictors. A sensitivity analysis adjusting the baseline model for the presence of oral alkali therapy was also performed.

All statistical analyses were performed using SAS/STAT software, version 9.4 (Cary, NC, USA). *P* values < 0.05 were considered statistically significant. All methods were carried out in accordance with relevant guidelines and regulations.

## Results

### Study cohort and characteristics

**Fig. 1:**
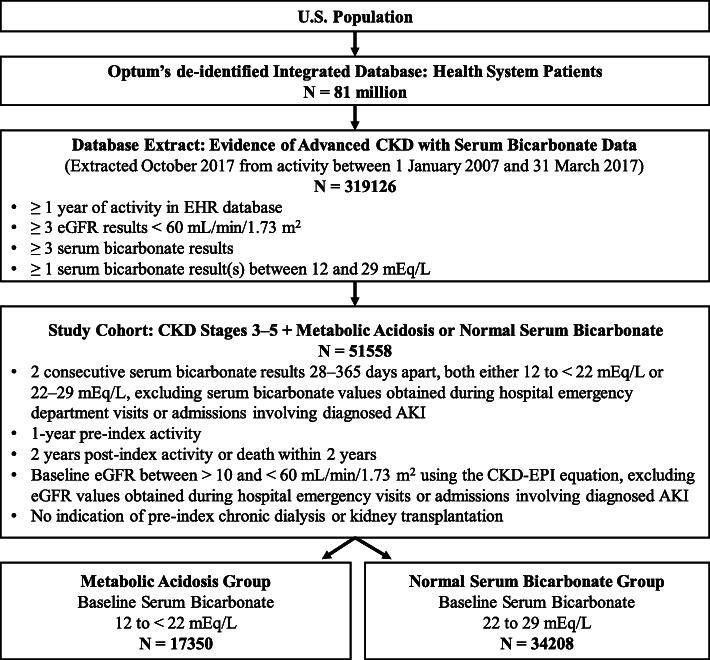
Patient selection flow diagram. Investigators had full access to the database extract but no direct access to the Optum database. Abbreviations: AKI, Acute Kidney Injury; CKD, chronic kidney disease; CKD-EPI, Chronic Kidney Disease Epidemiology Collaboration; eGFR, estimated glomerular filtration rate; EHR, Electronic Health Records

The Optum database contained 81 million patient records, of which 319,126 met the criteria for inclusion in the database extract. Within this database extract, we identified a study cohort of 51,558 patients who met data sufficiency requirements, had stage 3‒5 CKD with no indication of dialysis or transplant, and who qualified for inclusion in the metabolic acidosis group (N = 17,350) or normal serum bicarbonate group (N = 34,208) **(**Fig. 1). The distribution of the study population by baseline serum bicarbonate is provided in Additional file 4.

Individuals in the metabolic acidosis group had lower levels of serum bicarbonate (mean 19.7 vs. 26.1°mEq/L), were younger (mean age 70.3 vs. 74.3 years), were more likely to be African American (15 % vs. 7 %), had more advanced CKD (baseline eGFR 37.2 vs. 43.2°mL/min/1.73°m^2^), had a greater burden of comorbidities (coronary artery disease, diabetes, hypertension, heart failure, peripheral vascular disease, and a higher comorbidity burden as measured by the Charlson Comorbidity Index), and had higher mean urine ACR (277 mg/g vs. 127 mg/g) (all *P* < 0.001) (Table 1). Patient characteristics of the primary analysis cohort (excludes patients with missing ACR data) are shown in Additional file 5.

**Table 1:** Study Cohort Characteristics

	Total Study Cohort N = 51,558	Metabolic Acidosis Group N = 17,350	Normal Serum Bicarbonate Group N = 34,208	*P* value
Sex, n (%)				
Female	27,094 (53)	9,011 (52)	18,083 (53)	0.047
Male	24,464 (47)	8,339 (48)	16,125 (47)	0.047
Age, mean ± SD	72.9 ± 11.5	70.3 ± 13.3	74.3 ± 10.3	< 0.001
Race, n (%)				
African American	5,128 (10)	2,585 (15)	2,543 (7)	< 0.001
Asian	996 (2)	398 (2)	598 (2)	< 0.001
Caucasian	42,055 (82)	12,866 (74)	29,189 (85)	< 0.001
Other/unknown	3,379 (7)	1,501 (9)	1,878 (5)	< 0.001
Region, n (%)				
Midwest	30,683 (60)	9,359 (54)	21,324 (62)	< 0.001
Northeast	2,603 (5)	1,175 (7)	1,428 (4)	< 0.001
Other/unknown	586 (1)	227 (1)	359 (1)	0.009
South	14,107 (27)	5,329 (31)	8,778 (26)	< 0.001
West	3,579 (7)	1,260 (7)	2,319 (7)	0.041
Baseline labs, mean ± SD				
Serum bicarbonate, mEq/L	24.0 ± 3.6	19.7 ± 1.1	26.1 ± 2	< 0.001
eGFR, mL/min/1.73 m^2^	41.2 ± 12.1	37.2 ± 13.3	43.2 ± 10.9	< 0.001
ACR, urinary, mg/g	190 ± 554	277 ± 692	127 ± 414	< 0.001
CKD stage, n (%)				
Stage 3a	22,431 (44)	5,719 (33)	16,712 (49)	< 0.001
Stage 3b	19,081 (37)	5,987 (35)	13,094 (38)	< 0.001
Stage 4	8,736 (17)	4,747 (27)	3,989 (12)	< 0.001
Stage 5, non-dialysis	1,310 (3)	897 (5)	413 (1)	< 0.001
Comorbidities/conditions, n (%)				
Hypertension	31,761 (62)	12,879 (74)	18,882 (55)	< 0.001
Diabetes	16,168 (31)	7,391 (43)	8,777 (26)	< 0.001
Coronary artery disease	14,329 (28)	6,249 (36)	8,080 (24)	< 0.001
Peripheral vascular disease	10,052 (19)	5,038 (29)	5,014 (15)	< 0.001
Heart failure	10,029 (19)	5,119 (30)	4,910 (14)	< 0.001
CCI, weighted non-renal score, mean ± SD	2.3 (2.7)	3.5 (3.1)	1.7 (2.3)	< 0.001
ACE and ARB prescription, n (%)	12,041 (23)	5,110 (29)	6,931 (20)	< 0.001
Alkali treatment, n (%)	807 (2)	461 (3)	346 (1)	< 0.001
Additional baseline labs, mean ± SD				
Serum albumin, g/dL	3.7 ± 0.6	3.5 ± 0.7	3.9 ± 0.5	< 0.001
Serum calcium, corrected mg/dL^a^	9.3 ± 0.6	9.3 ± 0.7	9.4 ± 0.5	< 0.001
Hemoglobin, g/dL	12.2 ± 2.0	11.3 ± 2.1	12.6 ± 1.8	< 0.001
Serum potassium, mEq/L	4.4 ± 0.6	4.5 ± 0.7	4.4 ± 0.5	< 0.001

*Abbreviations*: *ACE *Angiotensin-converting enzyme inhibitor, *ACR *Albumin-creatinine ratio, *ARB *Angiotensin II receptor blockers, *CCI *Charlson Comorbidity Index, *CKD *Chronic kidney disease, *eGFR *Estimated glomerular filtration rate, *SD *Standard deviation^a^ Conversion factors for units, serum calcium mg/dL to mmol/L x 0.2495

### Renal outcomes

#### Unadjusted analyses

**Fig. 2:**
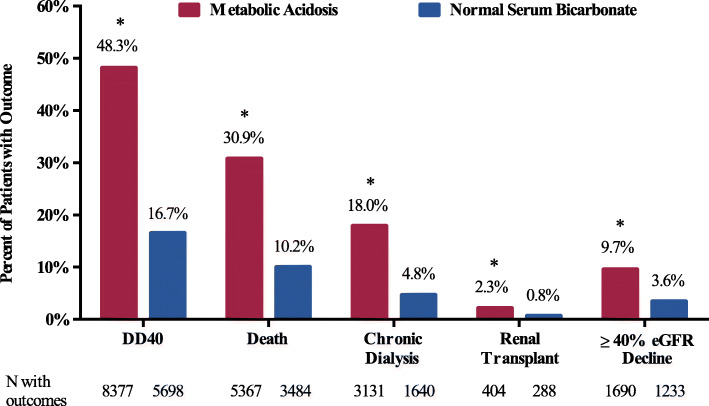
Two-year adverse renal and fatal outcomes among patients with chronic kidney disease with or without baseline metabolic acidosis (unadjusted analysis). The analysis included patients from the extended cohort: metabolic acidosis group, N = 17,350; normal serum bicarbonate group, N = 34,208). Abbreviation: DD40, ≥ 40 % eGFR decline, renal replacement therapy, or all-cause mortality; eGFR, estimated glomerular filtration rate. *P < 0.001: metabolic acidosis group vs. normal serum bicarbonate group

Patients with metabolic acidosis at baseline were more likely to experience adverse kidney outcomes or die compared with patients with normal serum bicarbonate. Over a period of up to 10 years (median 3.9 years), 12,861 patients (74.1 %) with metabolic acidosis vs. 17,602 (51.5 %) patients with normal serum bicarbonate at baseline experienced DD40 events. Over a 2-year period, all measured renal outcomes were significantly higher among patients with metabolic acidosis vs. normal serum bicarbonate, with an approximately 3-fold higher incidence of DD40 events (48.3 % vs. 16.7 %), death (31 % vs. 10 %), initiation of chronic dialysis (18 % vs. 5 %), and kidney transplant (2.3 % vs. 0.8 %) and an approximately 2-fold higher incidence of a ≥ 40 % decline in eGFR (10 % vs. 4 %) (*P* < 0.001 for all outcomes) (Fig. 2).

**Fig. 3:**
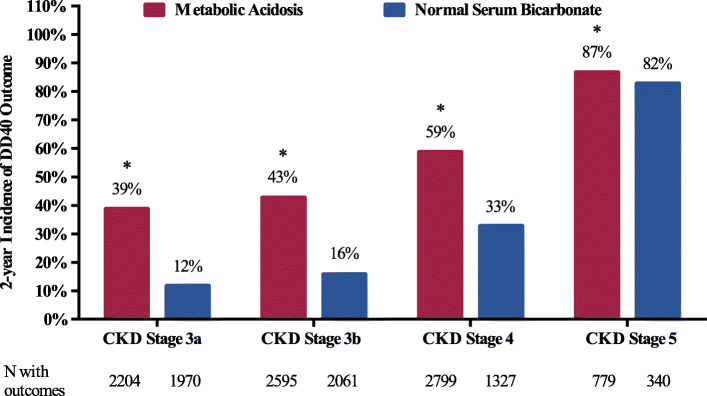
Two-year incidence of the composite outcome of DD40 by CKD stage (unadjusted analysis). The analysis included patients from the extended cohort: metabolic acidosis group, N = 17,350; normal serum bicarbonate group, N = 34,208). Abbreviations: CKD, chronic kidney disease; DD40, ≥ 40 % eGFR decline, renal replacement therapy, or all-cause mortality. *P < 0.001, **P < 0.05, metabolic acidosis group vs. normal serum bicarbonate group

The incidence of DD40 events at 2 years increased with CKD severity in the metabolic acidosis group and the normal serum bicarbonate group. Within each CKD subgroup, patients with metabolic acidosis had significantly higher rates of DD40 vs. patients with normal serum bicarbonate (CKD stage 3a: 39 % vs. 12 %, *P* < 0.001; CKD stage 3b: 43 % vs. 16 %, *P* < 0.001; CKD stage 4: 59 % vs. 33 %, *P* < 0.001; CKD stage 5: 87 % vs. 82 %, *P* = 0.03) (Fig. 3).

#### Analyses adjusted for potential confounders

The effects of selected covariates on the risk (evaluated using Cox proportional hazards models over ≤ 10 years) and odds (evaluated using a logistic regression model over ≤ 2 years) of a DD40 outcome are shown in Table 2. In all models, serum bicarbonate was a significant predictor of DD40. Over a ≤ 10-year period, each 1-mEq/L increase in serum bicarbonate was associated with a 7.4 % decrease in the risk of a DD40 outcome (hazard ratio [HR]: 0.926; 95 % CI, 0.922–0.930; *P* < 0.001) after controlling for age, sex, race, eGFR, pre-existing diabetes, hypertension, heart failure, CCI score, and log ACR. Serum bicarbonate was also independently associated with the 2-year DD40 outcome. Over a ≤ 2-year period, each 1-mEq/L increase in serum bicarbonate was associated with a 13 % decrease in the odds of a DD40 outcome (odds ratio [OR]: 0.873; 95 % CI, 0.866–0.879; *P* < 0.001) after controlling for age, sex, race, eGFR, pre-existing diabetes, hypertension, heart failure, CCI score, and log ACR.

**Table 2:** Effect of Selected Covariates on DD40

Covariate	Cox Proportional Hazards Model		Logistic Regression Model	
	**Hazard Ratio (95 % CI), DD40 up to 10 years**	***P value***^a^	**Odds Ratio (95 % CI), DD40 within 2 years**	***P value***^a^
Continuous Variables				
Serum bicarbonate, per 1 mEq/L increase	0.926 (0.922‒0.930)	< 0.001	0.873 (0.866‒0.879)	< 0.001
Age, per 1-year increase	1.005 (1.003‒1.006)	< 0.001	1.000 (0.998‒1.002)	0.846
Log ACR, per 1 mg/g increase	1.160 (1.150‒1.170)	< 0.001	1.209 (1.190‒1.229)	< 0.001
eGFR, per 1 mL/min/1.73 m^2^ increase	0.984 (0.983‒0.985)	< 0.001	0.967 (0.965‒0.969)	< 0.001
Categorical Variables				
Male	1.035 (1.006‒1.065)	0.017	1.197 (1.135‒1.263)	< 0.001
Race: African American (vs. Caucasian)	1.325 (1.268‒1.384)	< 0.001	1.436 (1.321‒1.560)	< 0.001
Race: Asian (vs. Caucasian)	0.886 (0.802‒0.978)	0.016	0.845 (0.704‒1.013)	0.068
Race: Other/unknown (vs. Caucasian)	1.078 (1.022‒1.138)	0.006	1.094 (0.989‒1.210)	0.079
Diabetes	1.034 (1.000‒1.069)	0.049	0.924 (0.868‒0.983)	0.013
Heart Failure	1.489 (1.437‒1.543)	< 0.001	1.828 (1.711‒1.953)	< 0.001
Hypertension	0.896 (0.863‒0.931)	< 0.001	0.938 (0.871‒1.011)	0.094
CCI score: 1 vs. 0	1.034 (0.982‒1.090)	0.204	1.157 (1.045‒1.281)	0.005
CCI score: 2 vs. 0	1.115 (1.060‒1.173)	< 0.001	1.212 (1.100‒1.334)	< 0.001
CCI score: ≥ 3 vs. 0	1.371 (1.310‒1.434)	< 0.001	1.739 (1.597‒1.895)	< 0.001

Abbreviations: ACR, albumin-creatinine ratio; CCI, Charlson Comorbidity Index; CI, confidence interval; DD40, a ≥ 40 % decline in eGFR, renal replacement therapy, or all-cause mortality; eGFR, estimated glomerular filtration rate

^a^ Statistically significant at *P* < 0.05

**Fig. 4:**
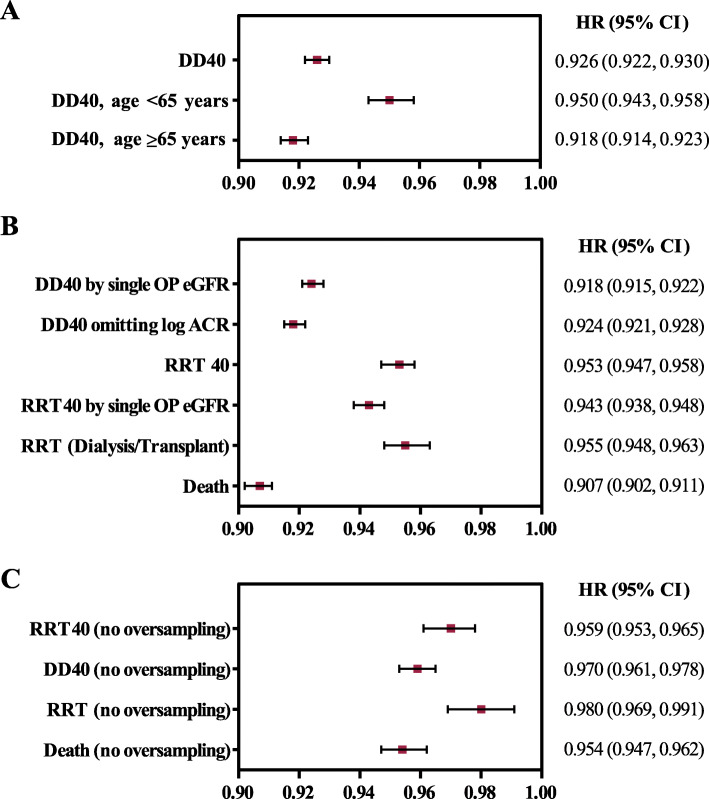
Adjusted risk of all-cause mortality and CKD progression per 1 mEq/L increase in serum bicarbonate. **a** Cox proportional hazards ratios for DD40 in the extended cohort (patients with no missing ACR data), with subgroup analysis by age group (< 65 and ≥ 65 years of age). **b** Cox proportional hazards ratios for secondary outcomes (RRT40, RRT, and all-cause mortality) and sensitivity analyses (DD40 and RRT40 by the first single outpatient eGFR value representing a decline of ≥ 40 % from baseline eGFR). **c** Cox proportional hazards ratios for primary and secondary outcomes in the primary analysis cohort (no missing ACR data). All analyses were adjusted for all covariates other than the analysis omitting ACR in Panel **b**, and all analyses in Panel **c**, which included all covariates other than ACR. Abbreviations: ACR, albumin-creatinine ratio; DD40, all-cause mortality, renal replacement therapy, or a ≥ 40 % decline in eGFR; HR, hazard ratio; LCL, lower confidence limit; RRT, renal replacement therapy; RRT40 = renal replacement therapy or a ≥ 40 % decline in eGFR; single OP eGFR, analysis in which the component of a ≥ 40 % decline in eGFR is established by a single outpatient eGFR measurement rather than an average eGFR during a 90-day period; UCL, upper confidence limit

Each 1-mEq/L increase in baseline serum bicarbonate was also associated with a 4.7 %, 4.5 %, and 9.3 % decrease in the risk of RRT40 (HR: 0.953; 95 % CI, 0.947–0.958; *P* < 0.001), RRT (HR: 0.955; 95 % CI, 0.948–0.963; *P* < 0.001), and all-cause mortality (HR: 0.907; 95 % CI, 0.902–0.911; *P* < 0.001) over ≤ 10 years, respectively (Fig. 4b).

#### Subgroup and sensitivity analyses

The significance of serum bicarbonate as a predictor of DD40 was sustained in subgroups analyzed by age (Fig. 4 a). Serum bicarbonate remained a significant independent predictor of DD40, RRT40, RRT, and all-cause mortality in the extended cohort (included patients who had missing values for urine ACR) and in the model using the alternative definition of eGFR decline (Fig. 4b). In addition, serum bicarbonate remained a significant independent predictor (*P* < 0.001) of DD40, RRT40, RRT, and all-cause mortality when assessed in a sensitivity analysis cohort constructed without the oversampling of metabolic acidosis (Fig. 4 c). With respect to our sensitivity analysis considering the receipt of oral alkali therapy, we found that few patients (2 % of the study cohort) were receiving oral alkali, and the results of our model were unchanged (Additional file 6).

## Discussion

In this large longitudinal cohort study of more than 50,000 community-based individuals with non-dialysis-dependent stage 3‒5 CKD, we found that a higher baseline serum bicarbonate level was independently associated with a lower risk of CKD progression, kidney failure, and all-cause mortality, when considered alone or as a composite outcome.

Each 1-mEq/L increase in serum bicarbonate was associated with a significant 7.4 % reduction in the risk of the DD40 outcome over a median follow-up of almost 4 years as well as a significant 13 % reduction in the odds of the DD40 outcome over a 2-year period. Previous studies examining the association between serum bicarbonate levels and CKD progression have found conflicting results, potentially due to differences in sample sizes, patient populations, baseline risks of end-stage kidney disease, and duration of follow-up as well as the definition and number of events, the prevalence and severity of metabolic acidosis, and the degree of adjustment for covariates, all of which influence power and effect size [6, 8, 17, 18]. Our ability to detect an association between small incremental increases in serum bicarbonate level and clinical outcomes was enabled by the large sample size and the high frequency of events in our study.

The results of our study are supported by findings from several clinical trials in which treatment of metabolic acidosis using various interventions led to reductions in adverse renal outcomes. One of these studies was a multicenter, multinational, placebo-controlled trial [19]. The other studies were open-label with standard-of-care controls, of which three were conducted at a single center and one was conducted at multiple centers in one country; interventions included a very low protein diet [20] or sodium bicarbonate [21–23].

Despite clinical practice guideline recommendations by Kidney Disease: Improving Global Outcomes (KDIGO) to treat metabolic acidosis in patients with CKD with oral bicarbonate supplementation, few patients with metabolic acidosis receive alkali treatment. For example, in the Chronic Renal Insufficiency Cohort (CRIC) study, only 2.7 % of patients with serum bicarbonate levels ≤ 22 mEq/L were receiving alkali treatment at baseline [6], and in the phase 3 veverimer study, less than < 10 % of patients with mean serum bicarbonate levels of 17.1 mEq/L were receiving sodium bicarbonate treatment at baseline [19]. From a clinical perspective, our findings suggest that metabolic acidosis should be treated more aggressively and patients should receive appropriate dietary counseling [24, 25] and pharmacological treatment when appropriate.

Our study has several strengths. To our knowledge, this is the largest longitudinal community-based cohort in which renal outcomes have been explored in patients with CKD. While generalizability to U.S. community populations cannot be guaranteed, it is strengthened by the inclusion of more than 50,000 patients with baseline eGFR < 60 mL/min/1.73 m^2^ as well as concurrent serum bicarbonate testing from a pool of over 80 million patients across every U.S. State and Puerto Rico, not limited by insurance type or insurance status. We employed conservative definitions to increase the specificity of our outcome measures by excluding eGFR and serum bicarbonate values from hospital care involving diagnoses of acute kidney injury, which enabled improved identification of CKD and chronic metabolic acidosis. Additionally, our measurement of eGFR decline by averaging results over a 90-day period was more conservative than that employed in similar analyses using single laboratory test results, in that it largely avoided defining outcomes based on a single, potentially spurious value [6]. The robustness of our findings is underscored by the consistent findings from sensitivity analyses and across subgroups defined by age, across CKD stages, and using different definitions of CKD progression. Not unexpectedly, the association of metabolic acidosis on adverse outcomes among patients with stage 3‒4 CKD was greater than for stage 5 CKD, where CKD progression or death during a 2-year timeframe is likely inevitable. Furthermore we examined the traditional end points of RRT and all-cause mortality, but also the newer, validated CKD progression end point of ≥ 40 % eGFR decline [12, 16], all of which likely increased the power of our study to detect associations between serum bicarbonate and adverse renal outcomes and mortality as well as more precisely estimate the risk of metabolic acidosis.

Our study also has several limitations. Because of its retrospective and observational nature, it is possible that residual confounding exists that we were unable to adjust for in our analyses. Identification of dialysis initiation in U.S. EHR data is limited by the lack of data from the specialized providers of most dialysis care. Although we addressed this limitation by defining dialysis based on an internal validation that cross-referenced EHR data and medical insurance claims, it should be noted that this definition has not been externally validated. Lastly, we only considered laboratory values at baseline, and any time-dependent effects were not captured in our models.

## Conclusions

Among patients with non-dialysis-dependent stage 3‒5 CKD, low levels of serum bicarbonate within the range of metabolic acidosis are independently associated with increased risk of DD40 (reduction in eGFR ≥ 40 %, RRT, or all-cause mortality), RRT40 (RRT or reduction in eGFR ≥ 40 %), and the individual outcomes of RRT and all-cause mortality. These findings are consistent with findings from recent clinical trials. Taken together, our study, combined with evidence from randomized, controlled trials, indicate that a low serum bicarbonate level may be an important modifiable risk factor for CKD progression and mortality. Efforts to improve disease awareness and treatment of metabolic acidosis in patients with CKD are urgently needed.

## Supplementary Information

Additional file 1. Summary of data sources and definitions.

Additional file 2. Validation study on identification of dialysis initiation.

Additional file 3. ICD-9-CM and ICD-10-CM Diagnosis Codes for Comorbidities and Outcomes.

Additional file 4. Patient distribution by baseline serum bicarbonate category.

Additional file 5. Patient characteristics of the primary cohort^a^.

Additional file 6. Effect of Selected Covariates on DD40: Cox Proportional Hazards Model Sensitivity Analysis Adding Adjustment for Prescription for Alkali Therapy. (N = 24,256 contributing to analysis)

## Data Availability

The data that support the findings of this study are available from [Optum] but restrictions apply to the availability of these data, which were used under license for the current study, and so are not publicly available.

## Abbreviations

ACR: Albumin-creatinine ratio
CCI: Charlson Comorbidity Index
CI: Confidence interval
CKD: Chronic kidney disease
CKD-EPI: Chronic Kidney Disease Epidemiology Collaboration
CRIC: Chronic Renal Insufficiency Cohort
DD40: a >40% decline in eGFR, renal replacement therapy, or all-cause mortality
DRG: Diagnosis-related group
eGFR: Estimated glomerular filtration rate
EHR: Electronic Health Record
HIPAA: Health Insurance Portability and Accountability Act
HR: Hazard ratio
ICD-9-CM: International Classification of Diseases, Ninth Revision, Clinical Modification
ICD-10-CM: International Classification of Diseases, Tenth Revision, Clinical Modification
KDIGO: Kidney Disease: Improving Global Outcomes
OR: Odds ratio
RRT: Renal replacement therapy
RRT40: Decline in eGFR ≥40%
U.S.: United States
