# Supplementary material for: Metabolic acidosis is associated with increased risk of adverse kidney outcomes and mortality in patients with non-dialysis dependent chronic kidney disease: an observational cohort study
Source: BMC Nephrol. 2021 May 19;22:185. doi: 10.1186/s12882-021-02385-z (PMC8136202; doi:10.1186/s12882-021-02385-z)
Supplement: Supplementary file 1 — Summary of data sources and definitions. [file 12882_2021_2385_MOESM1_ESM.docx]

## Additional File 1. Summary of Data Sources and Definitions

| **Variable** | **How Defined** | **Source(s)** | **Lowest Valid** | **Highest Valid** |
| --- | --- | --- | --- | --- |
| Sex | Dichotomous | Patient-level variable from vendor |  |  |
| Age | Years | Year of birth; all years before 1928 normalized to 1928 due to privacy concerns |  |  |
| Race | Optum-defined categories | Patient-level variable from vendor |  |  |
| Region | U.S. Census Regions | Patient-level variable from vendor |  |  |
| Baseline serum bicarbonate (mEq/L) | The first of the 2 consecutive, valid, consistent values (i.e., both 12 to < 22 or 22 to 29) separated by 28-365 days that establish index date | Laboratory tests not collected during inpatient hospitalizations or emergency department visits with a diagnosis of acute kidney injury | 8 | 40 |
| Baseline eGFR (mL/min/1.73 m^2^) | The mean of eGFR values (CKD-EPI) during the 90 days preceding the last eGFR test on or before the index date; multiple values per calendar day were averaged to contribute singly to this mean. | Serum creatinine laboratory tests not collected during inpatient hospitalizations or emergency department visits with a diagnosis of acute kidney injury | Serum creatinine > 0 | Serum creatinine < 20 |
| CKD^a^ |  |  |  |  |
| Stage 3a | eGFR 45 to < 60 mEq/L | Baseline eGFR |  |  |
| Stage 3b | eGFR 30 to < 45 mEq/L | Baseline eGFR |  |  |
| Stage 4 | eGFR 15 to < 30 mEq/L | Baseline eGFR |  |  |
| Non-dialysis CKD stage 5 | eGFR > 10 to < 15 mEq/L | Baseline eGFR |  |  |
| Comorbidities/conditions^b^ |  |  |  |  |
| Coronary artery disease | ≥ 1 during all available pre-index data | ICD-9 and ICD-10 diagnosis codes |  |  |
| Diabetes | ≥ 1 during all available pre-index data | ICD-9 and ICD-10 diagnosis codes |  |  |
| Heart failure | ≥ 1 during all available pre-index data | ICD-9 and ICD-10 diagnosis codes |  |  |
| Hypertension | ≥ 1 during all available pre-index data | ICD-9 and ICD-10 diagnosis codes |  |  |
| Peripheral vascular disease | ≥ 1 during all available pre-index data | ICD-9 and ICD-10 diagnosis codes |  |  |
| CCI, weighted^c^ | Single occurrence of diagnosis code per Quan (2005); weighted index score per Halfan (2002). | ICD-9 and ICD-10 diagnosis codes during 1-year pre-index period |  |  |
| Additional baseline labs^d^ |  |  |  |  |
| ACR, urinary (mg/g)^e^ | Laboratory value closest ≤ index date, using all available pre-index data | Urinary ACR, or conversions from protein-creatinine ratio or dipstick proteinuria results |  |  |
| Serum albumin (g/dL) | Laboratory value closest ≤ index date | Laboratory value as reported | 1 | 6 |
| Serum calcium, corrected (mg/dL) | Laboratory value closest ≤ index date, calculated as [total calcium + 0.8 * (4 - serum albumin)] using baseline serum albumin if available, otherwise as reported | Laboratory value as calculated or reported | 4 | 18 |
| Hemoglobin (g/dL) | Laboratory value closest ≤ index date | Laboratory value as reported | 6 | 19 |
| Serum potassium (mEq/L) | Laboratory value closest ≤ index date | Laboratory value as reported | 1 | 10 |

Abbreviations: ACR, albumin-creatinine ratio; CCI, Charlson Comorbidity Index; CKD, chronic kidney disease; eGFR, estimated glomerular filtration rate.

^a^Defined according to KSDIGO 2012 Clinical Practice Guideline for the Evaluation and Management of Chronic Kidney Disease [1].

^b^Comorbidities were identified as at least one occurrence of a diagnosis code in all available pre-index EHR data using coding algorithms shown in Additional file 3.

^c^CCI was evaluated as a single occurrence of an ICD-9 or ICD-10 diagnosis code in EHR records during the 12 months preceding the index date as defined by Quan et al (2005) [2] and was computed as a weighted index score using assigned weights developed by Halfon et al (2002) [3]. We express appreciation to the Manitoba Centre for Health Policy of the University of Manitoba for SAS macros based on these sources that we adapted to our dataset [4].

^d^For all laboratory tests, test date was based on sample collection date if available, otherwise the date the test result was reported. Data was restricted to tests no more than 1 year before the index date, except as noted.

^e^Urine protein-to-creatinine ratios were converted to ACR by dividing by 2.655 for women and 1.7566 for men [5]. Dipstick urine results were assigned estimated ACR values in mg/g as follows: -, negative = 9; “trace” = 43; Plus, plus one, 1, 1+ or + = 81; Plus 2, 2 plus, 2+ = 315; 3+, 4+ or similar = 1073. ACR was log-transformed in regression analyses due to skew.

**References**

1. KSDIGO 2012 Clinical Practice Guideline for the Evaluation and Management of Chronic Kidney Disease. Kidney Int. Suppl. 2013;3.
2. Quan H, Sundararajan V, Halfon P, et al. Coding algorithms for defining comorbidities in ICD-9-CM and ICD-10 administrative data. Med Care. 2005;43:1130-1139.
3. Halfon P, Eggli Y, van Melle G, Chevalier J, Wasserfallen JB, Burnand B. Measuring potentially avoidable hospital readmissions. J Clin Epidemiol. 2002;55:573-587.
4. University of Manitoba. Concept: Charlson Comorbidity Index. http://mchp-appserv.cpe.umanitoba.ca/viewConcept.php?conceptID=1098. Accessed June 13, 2019. SAS macros adapted to study dataset.

Inker LA, Levey AS, Pandya K, et al. Early change in proteinuria as a surrogate end point for kidney disease progression: an individual patient meta-analysis. Am J Kidney Dis. 2014;64(1):74-85.
