# Supplementary material for: Metabolic acidosis is associated with increased risk of adverse kidney outcomes and mortality in patients with non-dialysis dependent chronic kidney disease: an observational cohort study
Source: BMC Nephrol. 2021 May 19;22:185. doi: 10.1186/s12882-021-02385-z (PMC8136202; doi:10.1186/s12882-021-02385-z)
Supplement: Supplementary file 2 — Validation study on identification of dialysis initiation. [file 12882_2021_2385_MOESM2_ESM.docx]

## Additional File 2. Validation Study on Identification of Dialysis Initiation

In the U.S., most chronic dialysis treatments are provided in commercial dialysis centers operated by specialized companies, such as Fresenius Medical Care and DaVita Kidney Care, that maintain independent medical record systems. No major commercial chronic dialysis company has publicly reported participating in EHR data-sharing with Optum or similar services or integrating its EHR system with those of health systems where dialysis patients receive other medical care. This places limitations on retrospective renal studies using U.S. EHR data. Insurance claim databases do not have this limitation, because the insurer receives claims from all providers caring for a patient. Additionally, because the Optum dataset is de-identified, it was not possible to link the data from individual patients to their dialysis and transplant data in the United States Renal Data System (USRDS).

To address these limitations, we developed a proxy definition for initiation of dialysis using data points routinely populated in EHR systems, which we tested by comparing to medical claims indicators of dialysis in a subset of patients for which Optum provided concurrent data for EHR and for insurance claims. The definition used diagnosis codes from the Ninth and Tenth revisions of the International Statistical Classification of Diseases and Related Health Problems (ICD-9 and ICD-10, respectively), Current Procedural Terminology (CPT) codes, and Healthcare Common Procedural Coding System (HCPCS) codes, along with eGFR laboratory test results (in EHR data). Specifically, ICD-9, ICD-10, CPT, and HCPCS codes used by the USRDS to identify dialysis in claims data [3]. were used in conjunction with claims indicators, such as provider type and place of service to manually establish whether and when chronic dialysis was initiated. In EHR data, dialysis identifications using those diagnoses, CPT, and HCPCS codes plus a cutoff value of single eGFR ≤ 10 mL/min/1.73 m^2^ were compared to the claims data results of patients with concurrent EHR and claims data. After initial testing, we refined the definition slightly by lowering the eGFR cutoff from 10 mL/min/1.73 m^2^ to 9 mL/min/1.73 m^2^. The final definition identified chronic dialysis in EHR data at the first occurrence of any of the following indicators:

1. Any eGFR ≤9 mL/min/1.73m^2^ from a sample not collected during a hospital inpatient admission or emergency department visit
2. ICD-9 Diagnosis code 585.6, V45.11, V56.0, V56.1, V56.2, V56.31, V56.32, V56.8, or E87.91
3. ICD-10 Diagnosis code N18.6, Z99.2, Z49.31, Z49.01, Z49.02, Z49.31, Z49.32, or Z49.32
4. CPT Code 90918, 90919, 90920, 90921, 90922, 90923, 90924, 90925, 90935, 90937, 90940, 90945, 90947, 90951, 90952, 90953, 90954, 90955, 90956, 90957, 90958, 90959, 90960, 90961, 90962, 90963, 90964, 90965, 90966, 90967, 90968, 90969, 90970, 90989, 90993, 90997, or 90999
5. HCPCS codes G0317-G0327

This definition of chronic dialysis in EHR data demonstrated 90% sensitivity and 86% specificity when tested against dialysis incidence in the claims data, which we deemed to be acceptable for this definition.
