# Supplementary material for: Metabolic acidosis is associated with increased risk of adverse kidney outcomes and mortality in patients with non-dialysis dependent chronic kidney disease: an observational cohort study
Source: BMC Nephrol. 2021 May 19;22:185. doi: 10.1186/s12882-021-02385-z (PMC8136202; doi:10.1186/s12882-021-02385-z)
Supplement: Supplementary file 3 — ICD-9-CM and ICD-10-CM Diagnosis Codes for Comorbidities and Outcomes. [file 12882_2021_2385_MOESM3_ESM.docx]

## Additional File 3. ICD-9-CM and ICD-10-CM Diagnosis Codes for Comorbidities and Outcomes

| **Comorbidity** | **ICD-9-CM codes** | **ICD-10-CM codes** | **Diagnosis-Related Group Code** |
| --- | --- | --- | --- |
| Coronary artery disease | 410-414; V45.81; V45.82 | I20.0-I22.9; I24.0-I25.9; Z95.1; Z95.5; Z98.61 |  |
| Peripheral vascular disease | 440-444; 447; 451-453; 557 | E08.51; E08.52; E09.51; E09.52; E10.51; E10.52; E11.51; E11.52; E13.51; E13.52; I67.0; I70.0-I74.9; I77.0-I77.9; I79.0-I82.91; K55.0; K55.1; K55.8; K55.9; M31.8; M31.9 |  |
| Heart failure | 398.91; 402.01, 402.11, 402.91; 404.01, 404.03, 404.11, 404.13, 404.91, 404.93; 422; 425; 428; V42.1 | A18.84; I09.81; I11.0; I13.0; I13.2; I40.0-I43; I50.1-I50.9; Z48.21; Z48.280; Z94.1; Z94.3 |  |
| Diabetes | 250; 357.2; 362.0; 366.41 | E08.311-E08.36; E08.40; E08.42; E09.311-E09.36; E09.40; E09.42; E10.10-E13.9 |  |
| Hypertension | 362.11; 401-405; 437.2 | H35.031-H35.039, I10-I13.2, I15.0-I15.9, I67.4, N26.2 |  |
| Kidney transplantation | 00.91, 00.92, 00.93, 55.53, 55.61, 55.69, 996.81, V42.0 | T86.10, T86.11, T86.12, T86.13, T86.19, Z94.0, 0TY.00Z0, 0TY.00Z1, 0TY.00Z2, 0TY.10Z0, 0TY.10Z1, 0TY.10Z2 | 652 or 008 |
| Acute kidney injury | Between 584.5 and 584.9^a^ | Between N17.0 and N17.9 ^a^ |  |

^a^During dates of a hospital emergency room visit or hospital inpatient or observation admission.

**Reference**:

United States Renal Data System. 2018 USRDS annual data report: Epidemiology of kidney disease in the United States. National Institutes of Health, National Institute of Diabetes and Digestive and Kidney Diseases, Bethesda, MD, 2018.
