# Supplementary material for: Metabolic acidosis is associated with increased risk of adverse kidney outcomes and mortality in patients with non-dialysis dependent chronic kidney disease: an observational cohort study
Source: BMC Nephrol. 2021 May 19;22:185. doi: 10.1186/s12882-021-02385-z (PMC8136202; doi:10.1186/s12882-021-02385-z)
Supplement: Supplementary file 4 — Patient distribution by baseline serum bicarbonate category. [file 12882_2021_2385_MOESM4_ESM.docx]

## Additional File 4. Patient Distribution by Baseline Serum Bicarbonate Category

|  | Number (Percentage) of Patients |
| --- | --- |
| Baseline serum bicarbonate |  |
| 12 mEq/L | 99 (0.2) |
| 13 mEq/L | 116 (0.2) |
| 14 mEq/L | 166 (0.3) |
| 15 mEq/L | 262 (0.5) |
| 16 mEq/L | 454 (0.9) |
| 17 mEq/L | 755 (1.5) |
| 18 mEq/L | 1,403 (2.7) |
| 19 mEq/L | 2,378 (4.6) |
| 20 mEq/L | 4,347 (8.4) |
| 21 mEq/L | 7,370 (14.3) |
| 22 mEq/L | 1,577 (3.1) |
| 23 mEq/L | 2,439 (4.7) |
| 24 mEq/L | 3,682 (7.1) |
| 25 mEq/L | 4,941 (9.6) |
| 26 mEq/L | 5,825 (11.3) |
| 27 mEq/L | 5,956 (11.6) |
| 28 mEq/L | 5,575 (10.8) |
| 29 mEq/L | 4,213 (8.2) |

**References**

1. Tangri N, Grams ME, Levey AS, et al. Multinational assessment of accuracy of equations for predicting risk of kidney failure: a meta-analysis. JAMA. 2016;315(2):164-174. Supplemental Material e1.2.

United States Renal Data System. 2018 USRDS annual data report: Epidemiology of kidney disease in the United States. National Institutes of Health, National Institute of Diabetes and Digestive and Kidney Diseases, Bethesda, MD, 2018.
