# Supplementary material for: Metabolic acidosis is associated with increased risk of adverse kidney outcomes and mortality in patients with non-dialysis dependent chronic kidney disease: an observational cohort study
Source: BMC Nephrol. 2021 May 19;22:185. doi: 10.1186/s12882-021-02385-z (PMC8136202; doi:10.1186/s12882-021-02385-z)
Supplement: Supplementary file 5 — Patient characteristics of the primary cohorta. [file 12882_2021_2385_MOESM5_ESM.docx]

## Additional File 5. Patient Characteristics of the Primary Cohort^a^

|  | Priamry Cohort Total  N = 32,007 | Metabolic Acidosis  Group  N = 13,569 | Normal Serum Bicarbonate Group  N = 18,438 | *P* value |
| --- | --- | --- | --- | --- |
| Sex, n (%) |  |  |  |  |
| Female | 16,911 (53) | 7,155 (53) | 9,756 (53) | 0.747 |
| Male | 15,096 (47) | 6,414 (47) | 8,682 (47) | 0.747 |
| Age, mean ± SD | 72.6 ± 11.9 | 70.6 ± 13.3 | 74. ± 10.6 | < 0.001 |
| Race, n (%) |  |  |  |  |
| African American | 3,441 (11) | 2,014 (15) | 1,427 (8) | < 0.001 |
| Asian | 702 (2) | 319 (2) | 383 (2) | < 0.001 |
| Caucasian | 25,588 (80) | 10,025 (74) | 15,563 (84) | < 0.001 |
| Other/unknown | 2,276 (7) | 1,211 (9) | 1,065 (6) | < 0.001 |
| Region, n (%) |  |  |  |  |
| Midwest | 19,459 (61) | 7,478 (55) | 11,981 (65) | < 0.001 |
| Northeast | 1,661 (5) | 886 (7) | 775 (4) | < 0.001 |
| Other/unknown | 352 (1) | 170 (1) | 182 (1) | < 0.001 |
| South | 8,434 (26) | 4,143 (31) | 4,291 (23) | < 0.001 |
| West | 2,101 (7) | 892 (7) | 1,209 (7) | < 0.001 |
| Baseline labs, mean ± SD |  |  |  |  |
| Serum bicarbonate, mEq/L | 23.4 ± 3.7 | 19.7 ± 1.7 | 26.1 ± 2.0 | < 0.001 |
| eGFR, mL/min/1.73 m^2^ | 40.8 ± 12.3 | 37.5 ± 13.2 | 43.2 ± 10.9 | < 0.001 |
| ACR, urinary, mg/g | 190 ± 554 | 277 ± 692 | 127 ± 414 | < 0.001 |
| CKD stage, n (%) |  |  |  |  |
| Stage 3a | 13,556 (42) | 4,564 (34) | 8,992 (49) | < 0.001 |
| Stage 3b | 11,754 (37) | 4,721 (35) | 7,033 (38) | < 0.001 |
| Stage 4 | 5,880 (18) | 3,671 (27) | 2,209 (12) | < 0.001 |
| Stage 5, non-dialysis | 817 (3) | 613 (5) | 204 (1) | < 0.001 |
| Comorbidities/conditions, n (%) |  |  |  |  |
| Hypertension | 21,319 (67) | 10,549 (78) | 10,770 (58) | < 0.001 |
| Diabetes | 12,145 (38) | 6,329 (47) | 5,816 (32) | < 0.001 |
| Coronary artery disease | 9,737 (30) | 5,148 (38) | 4,589 (25) | < 0.001 |
| Peripheral vascular disease | 7,122 (22) | 4,242 (31) | 2,880 (16) | < 0.001 |
| Heart failure | 7,132 (22) | 4,308 (32) | 2,824 (15) | < 0.001 |
| CCI, weighted, mean (SD) | 2.7 ± 2.9 | 3.7 ± 3.2 | 2.0 ± 2.5 | < 0.001 |
| Additional baseline labs, mean ± SD |  |  |  |  |
| Serum albumin, g/dL | 3.7 ± 0.6 | 3.5 ± 0.7 | 3.8 ± 0.5 | < 0.001 |
| Serum calcium, corrected, mg/dL^b^ | 9.3 ± 0.6 | 9.3 ± 0.7 | 9.4 ± 0.5 | < 0.001 |
| Hemoglobin, g/dL | 12.0 ± 2.0 | 11.3 ± 2.1 | 12.6 ± 1.8 | < 0.001 |
| Serum potassium, mEq/L | 4.4 ± 0.6 | 4.5 ± 0.7 | 4.4 ± 0.5 | < 0.001 |

^a^The primary cohort consisted of patients who had no missing ACR data. Missing data were a consideration only for ACR, since the inclusion criteria required eGFR and serum bicarbonate results and a minimum of 1 year of patient activity in EHR data prior to the index date, during which the presence or absence of comorbidities could be assessed by diagnosis code. A total of 3,781 (22%) patients with metabolic acidosis and 15,770 (46%) patients with normal serum bicarbonate had missing ACR data.

Abbreviations: ACR, albumin-creatinine ratio; CCI, Charlson Comorbidity Index; CKD, chronic kidney disease; eGFR, estimated glomerular filtration rate; SD, standard deviation.

^b^Conversion factors for units, serum calcium mg/dL to mmol/L x 0.2495.
