# Supplementary material for: Metabolic acidosis is associated with increased risk of adverse kidney outcomes and mortality in patients with non-dialysis dependent chronic kidney disease: an observational cohort study
Source: BMC Nephrol. 2021 May 19;22:185. doi: 10.1186/s12882-021-02385-z (PMC8136202; doi:10.1186/s12882-021-02385-z)
Supplement: Supplementary file 6 — Effect of Selected Covariates on DD40: Cox Proportional Hazards Model Sensitivity Analysis Adding Adjustment for Prescription for Alkali Therapy. (N = 24,256 contributing to analysis) [file 12882_2021_2385_MOESM6_ESM.docx]

**Additional File 6. Effect of Selected Covariates on DD40: Cox Proportional Hazards Model Sensitivity Analysis Adding Adjustment for Prescription for Alkali Therapy. (N = 24,256 contributing to analysis)**

| Covariate | Hazard Ratio (95% CI) | *P* value | *P <0.05 |
| --- | --- | --- | --- |
| Continuous Variables |  |  |  |
| Serum bicarbonate, per 1-mEq/L increase | 0.92 (0.92,0.93) | <0.0001 | * |
| Age, per 1-year increase | 1.01 (1.00,1.01) | <0.0001 | * |
| eGFR, per 1 mL/min/1.73 m2 increase | 0.98 (0.98,0.99) | <0.0001 | * |
| Log ACR, per 1-unit increase | 1.15 (1.14,1.16) | <0.0001 | * |
| Categorical Variables |  |  |  |
| Male | 1.04 (1.01,1.07) | 0.02 | * |
| Race: African American (vs Caucasian) | 1.31 (1.25,1.38) | <0.0001 | * |
| Race: Asian (vs Caucasian) | 0.94 (0.84,1.05) | 0.26 |  |
| Race: Other/Unknown (vs Caucasian) | 1.06 (0.99,1.13) | 0.08 |  |
| Diabetes | 1.05 (1.01,1.09) | 0.01 | * |
| Heart Failure | 1.47 (1.42,1.53) | <0.0001 | * |
| Hypertension | 0.91 (0.87,0.95) | <0.0001 | * |
| CCI score: 1 vs 0 | 1.06 (1.00,1.13) | 0.07 |  |
| CCI score: 2 vs 0 | 1.14 (1.07,1.21) | <0.0001 | * |
| CCI score: ≥3 vs 0 | 1.38 (1.31,1.46) | <0.0001 | * |
| Alkali prescription | 1.14 (1.02,1.28) | 0.03 | * |
| ACR = albumin-creatinine ratio; CCI = Charlson Comorbidity Index; CI = Confidence Interval; eGFR=estimated glomerular filtration rate | | | |

Note: Patients having no records for prescribed or patient-reported medications in the pre-index period during which baseline covariates including alkali prescriptions were evaluated were considered to have missing prescription data and were excluded from this sensitivity analysis.
